# Supplementary figures and images for: Machine Learning Undercounts Reproductive Organs on Herbarium Specimens but Accurately Derives Their Quantitative Phenological Status: A Case Study of Streptanthus tortuosus
Source: Plants (Basel). 2021 Nov 16;10(11):2471. doi: 10.3390/plants10112471 (PMC8623300; doi:10.3390/plants10112471)

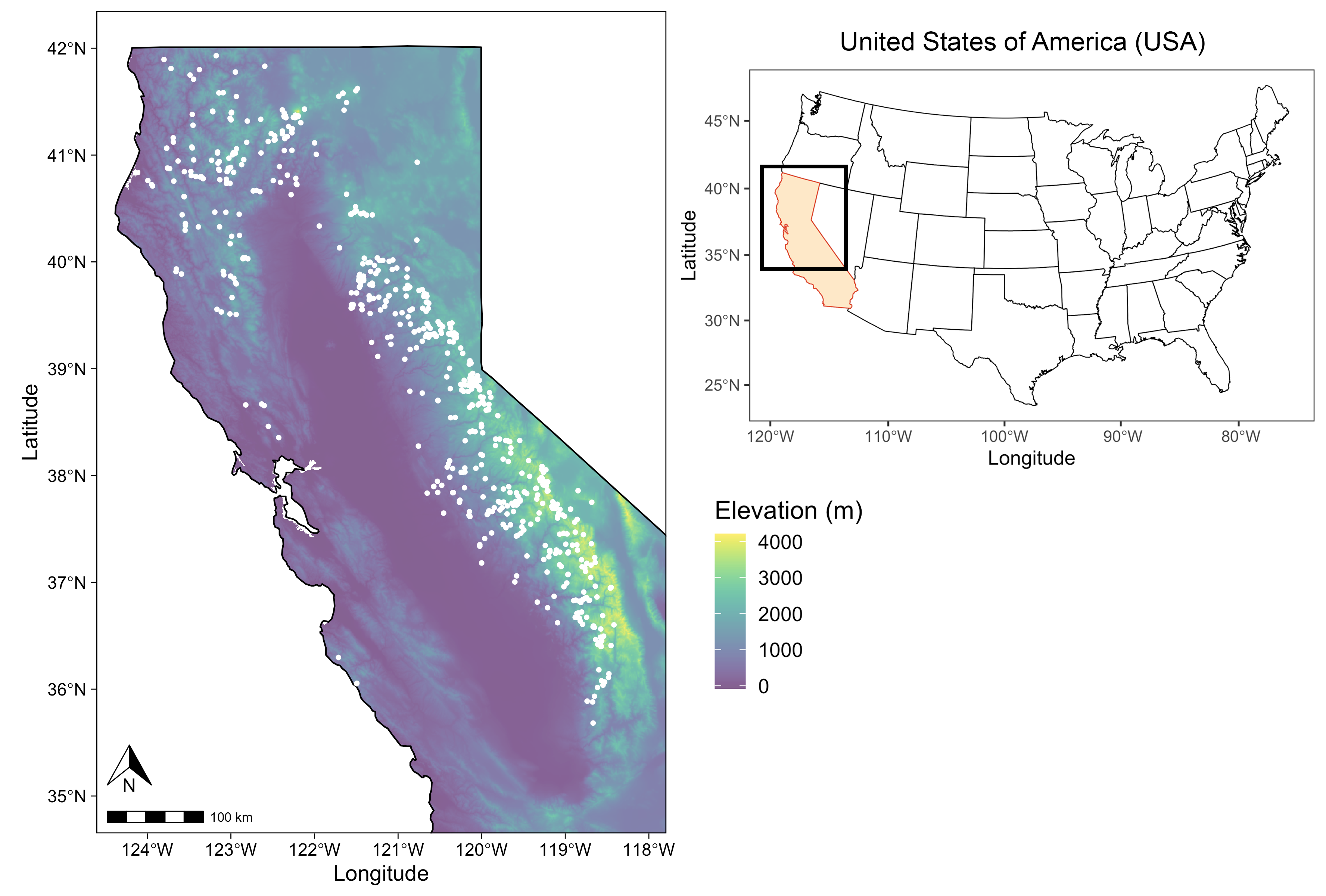

Supplement: Supplementary file 1 [file plants-10-02471-s001.zip › Supplement - Revision/Figure S1. Streptanthus_map.png]
